# Assessment Modalities for Lower Extremity Edema, Lymphedema, and Lipedema: A Scoping Review

**DOI:** 10.7759/cureus.55906

**Published:** 2024-03-10

**Authors:** Biura Markarian, Carel Toro, Karina Moreira, Sneha Polam, Neethu Mathew, Harvey N Mayrovitz

**Affiliations:** 1 Osteopathic Medicine, Nova Southeastern University Dr. Kiran C. Patel College of Osteopathic Medicine, Davie, USA; 2 Sports Medicine, Nova Southeastern University Dr. Kiran C. Patel College Of Osteopathic Medicine, Fort Lauderdale, USA; 3 Medical Education, Nova Southeastern University Dr. Kiran C. Patel College of Allopathic Medicine, Davie, USA

**Keywords:** edema diagnostic modalities, magnetic resonance imaging, computed tomography, ultrasound, bioimpedance spectroscopy, tissue dielectric constant, lymphoscintigraphy, lipedema, lower extremity edema, lymphedema

## Abstract

Lower extremity swelling may be broadly characterized as due to edema, lymphedema, or lipedema. Differentiation between these three conditions is important for providing appropriate treatment. This review analyzes and compares different clinical diagnostic modalities for these conditions, with the aim of assisting in the process of choosing the most appropriate diagnostic modality by highlighting the advantages and limitations of each.

Using the Preferred Reporting Items for Systematic Reviews and Meta-Analyses (PRISMA) guidelines for a systematic search of peer-reviewed literature, the following diagnostic methods for lower extremity swelling were investigated: (1) ultrasound (US), (2) lymphoscintigraphy (LSG), (3) computed tomography (CT), (4) bioimpedance spectroscopy (BIS), (5) tissue dielectric constant (TDC), and (6) magnetic resonance imaging (MRI), including magnetic resonance lymphangiography (MRL). The databases used in the search were PubMed, ProQuest, CINAHL Complete, Web of Science, Embase, and Biomedical Reference Collection. After retrieving 115 studies based on predetermined inclusion criteria, a total of 31 studies were critically evaluated.

The main results indicate the following: duplex US is the modality of choice to initially identify lower extremity edema such as deep venous thrombosis (DVT) and venous reflux due to its high sensitivity and specificity. CT venography of the lower extremity appears to bethe preferred option for gynecologic cancer patients with lower extremity swelling post-treatment, as it measures subcutaneous tissue volumes to look for DVTs, lymphoceles, and cancer recurrence. TDC is a recommended modality for a variety of conditions, including edema and lymphedema, in part, due to its noninvasive localized assessment capabilities and ease of use. LSG emerges as an effective imaging modality for lymphedema characterization with minimal invasiveness and high sensitivity and specificity. BIS is widely used to identify and monitor lower extremity lymphedema but has been reported to have low sensitivity and lacks the ability to account for changes in tissue composition such as fibrosis. US and MRL are favored for lipedema diagnosis, with MRL providing comprehensive anatomical and functional insights, albeit with cost and accessibility limitations compared to US. While CT, MRI, US, and TDC are all useful for differentiating lymphedema from lipedema, MRI is the preferred modality due to its anatomical and functional diagnostic capabilities. However, US is a pragmatic alternative for use with obese patients or when MRI is not an option.

## Introduction and background

Swelling of one or both lower extremities is a common condition with multiple possible causes ranging from minor venous valve dysfunction [1] to congestive heart disease [2]. The swelling may occur acutely if triggered by trauma, over time as primary lymphedema, or following gynecological surgery, urological surgery, prostatic surgery, or other interventions that affect the lymphatic system [3,4]. The characteristics of the swelling may differ depending on the cause, duration, and associated treatments. Based on the underlying cause, there are three broad categories that may be used to characterize lower extremity swelling: edema, lymphedema, and lipedema.

Lower extremity edema refers to swelling that occurs when capillary filtration into the interstitium exceeds, which can be reabsorbed or drained via the lymphatic system, resulting in interstitial fluid accumulation [5]. Causes include deep vein thrombosis (DVT), lymphatic insufficiency, infection, trauma, and many long-term medical conditions [6]. When looking at this lengthy differential diagnosis, dividing the causes between unilateral and bilateral lower extremity edema proves useful [7]. Some causes fall into either category [1]. When the edema presents unilaterally, a very urgent cause could be DVT [7]. This is more likely if the patient has risk factors such as recent surgery, long periods of inactivity, and/or hospitalization, combined with erythema and tenderness in the calf [1]. Other unilateral causes include superficial thrombophlebitis and cellulitis. When lower extremity edema presents bilaterally, one common cause is dependent edema, which results from episodes of prolonged sitting or standing, which allows for venous blood to pool. Heart failure, pulmonary hypertension, pregnancy, cirrhosis, kidney disease, and medications, such as calcium channel blockers, gabapentin or pregabalin, non-steroidal anti-inflammatory drugs, oral contraceptives, steroids, and thiazolidinediones, can also cause bilateral swelling of the lower extremities [1]. Lymphedema and lipedema can cause either unilateral or bilateral lower extremity edema (discussed below), as well as venous insufficiency, such as venous hypertension, chronic venous insufficiency, and post-thrombotic syndrome. Distinguishing among these possible causes may be achieved with physical examination in some cases, but diagnostic testing is often required [1].

Lymphedema is a progressive disease characterized by lymph fluid accumulation due to exceeding physiologic lymphatic drainage capacity, resulting in swelling in different body parts, especially in the extremities [7]. It usually initially presents as a painless, rubbery swelling that may continue into the dorsum of the foot and toes. Lymphedema is classified as either primary or secondary. Developmental malformations (aplasia, hypoplasia, hyperplasia) of the lymphatic system in the channels, nodes, or both, cause primary lymphedema, which is rare [8]. Secondary lymphedema, however, is more common, and results from underlying conditions such as infection, trauma, cancer and its treatment, obesity, and radiation causing injury or obstruction to the lymphatic system [9,10]. The most common etiology of secondary lymphedema worldwide is lymphatic filariasis, caused 90% of the time by the nematode *Wuchereria bancrofti*, which causes acute febrile episodes in addition to the deformity in lower extremities [11]. In the United States, the most common cause of lymphedema is surgical resection of lymph nodes after cancer treatment [10]. Depending on the stage, signs and symptoms may include soft, pitting edema, hyperkeratosis (thickening of the skin’s outer layer), lymphangioma (benign, fluid-filled cysts in the lymphatic system), and lymphorrhea (lymph leakage onto the skin) [9]. Early diagnosis and treatment are critical to prevent secondary complications such as cellulitis and to halt the progression to the chronic phase of the disease [9]. Diagnosing lymphedema requires a thorough history and physical examination, including the age of onset, medication, travel history, and family history [12].

Lipedema is a disorder presenting with localized accumulation of subcutaneous adipose tissue in the lower extremities, usually extending from the hips to ankles. Lipedema is bilateral and usually spares the feet, hands, and trunk [13]. It is accompanied by disproportionate pain, easy bruising, and tenderness, along with a physical aesthetic deformity [1,14]. The etiology of lipedema is unclear, but a genetic component is likely as it is often seen in females with a family history of lipedema and does not respond to weight loss or exercise [1,14]. It often presents at a young age, usually at the onset of puberty or other hormonal changes [1]. One hypothesis states that lipedema is an estrogen-regulated polygenetic disease that arises during female hormonal changes [15]. Due to its similarity in physical examination, lipedema is often misdiagnosed as obesity, especially since many clinicians are not familiar with the disease [16]. Lipedema can also be mistaken for lymphedema, but a major difference is lipedema almost always presents bilaterally while lymphedema may present as either bilateral or unilateral [16]. Lipedema can progress to lipolymphedema, which is characterized by a combination of both lipedema and lymphedema [17]. This is due to the increased pressure from the accumulation of fat in lipedema, which can compress the lymphatic vessels, further exacerbating lymphatic fluid buildup and contributing to swelling and tissue changes [17]. Because of the differences in etiology and treatments for each of these conditions, it is important to be able to differentiate between them clinically. This distinction should be made both initially and during treatment to reliably track progress in swelling and the limb’s tissue properties. For these purposes, several diagnostic methods have been described and utilized with varying degrees of complexity, cost, duration, and reliability. These methods include ultrasound (US) [6,18,19], lymphoscintigraphy (LSG) [20,21], computed tomography (CT) [22,23], bioimpedance spectroscopy (BIS) [24], tissue dielectric constant (TDC) [25], and magnetic resonance imaging (MRI). The purpose of this review is to describe these methods, explain their main application targets, and provide some guidance on the advantages and disadvantages of each method.

## Review

The following describes the details of the study eligibility criteria and search criteria used in this review.

Materials and methods

This scoping review utilized primary studies found via a search on the databases PubMed, ProQuest, CINAHL Complete, Web of Science, Embase, and Biomedical Reference Collection: Comprehensive Edition. The selection process was performed independently by five reviewers based on the inclusion and exclusion criteria.

Search Strategy

The inclusion and exclusion criteria were established prior to conducting the review. The inclusion criteria were as follows: (1) articles written in the English language, (2) peer-reviewed articles, (3) studies involving human adults aged 18 years and older, and (4) lower extremity only.

Identification and Selection of Studies

The following text words and search phrases were used: "diagnostic tools," "diagnosis," "diagnostic modality," "diagnos*," and "lower extremity edema," "lymphedema," or "lipedema." Later keyword searches were "ultrasound," "lymphoscintigraphy," "computed tomography," "bioimpedance spectroscopy," "tissue dielectric constant," or "magnetic resonance imaging." These keywords were combined in different ways with the purpose of expanding the searches.

**Figure 1:**
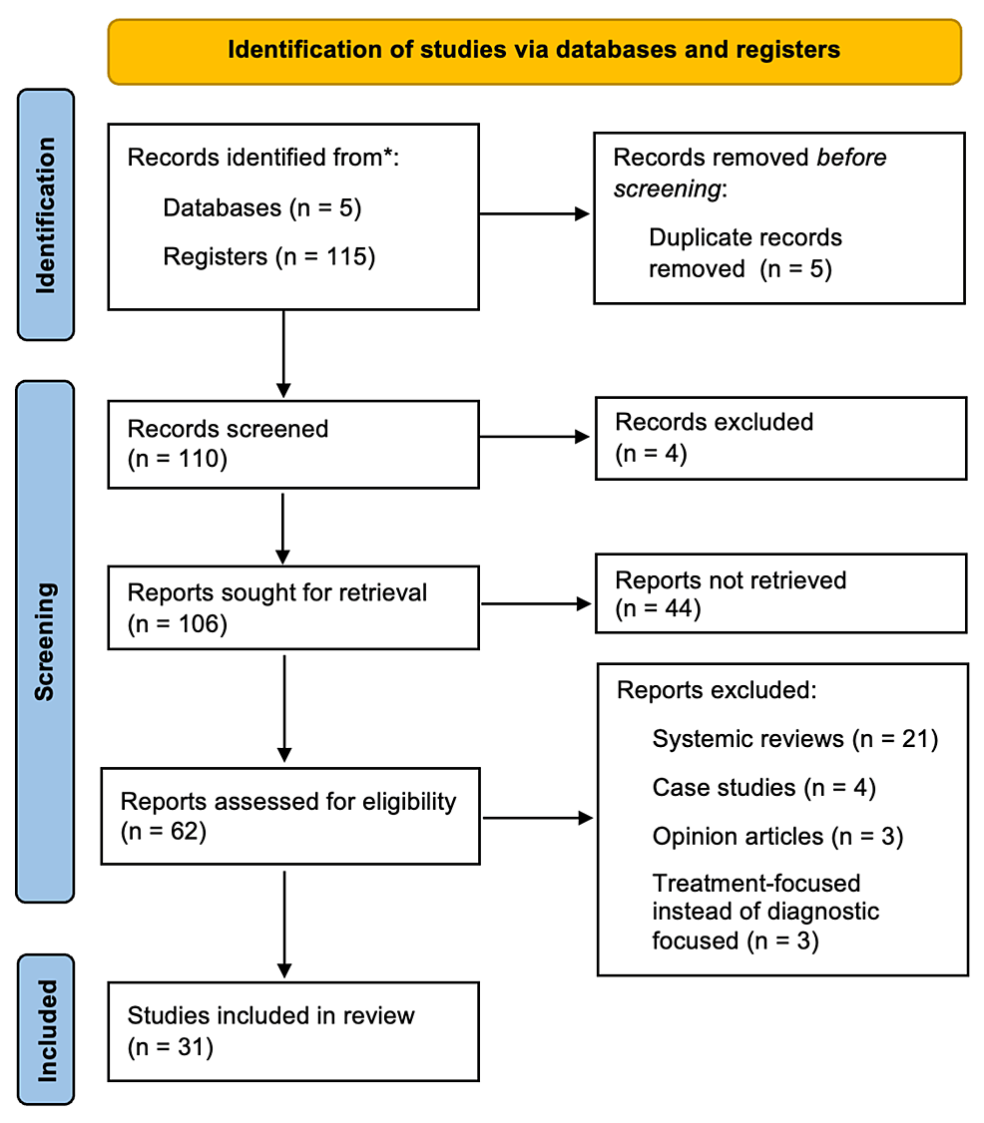
Search approach The diagram shows the Preferred Reporting Items for Systematic Reviews and Meta-Analyses (PRISMA) flowchart, which quantitatively depicts the study selection process and reasons for excluded reports.

The initial search yielded 115 articles, which were then screened based on the above search criteria. Five duplicates were removed, and 48 articles were subsequently removed that did not meet the inclusion criteria. The remaining 62 articles were further evaluated for eligibility and 31 were excluded based on the type of study conducted and various other reasons. This screening and selection process in more detail is depicted using the Preferred Reporting Items for Systematic Reviews and Meta-Analyses (PRISMA) flowchart in Figure 1. The Joanna Briggs Institute Appraisal Tools were used to assess the methodological quality, trustworthiness, including possible biases, and relevance of the 31 included studies [26]. After quality appraisal of these articles using the checklists for case-control, cohort, diagnostic test accuracy, qualitative research, and randomized controlled studies, no articles were excluded. This resulted in 31 articles for the final scoping review.

After screening and applying the inclusion criteria to the studies obtained from the relevant databases, two reviewers independently extracted relevant data from the included 31 studies and jointly created a data-charting sheet in Microsoft Excel (Microsoft Corporation, Redmond, WA). The sheet was organized and filled in with author, year of publication, country of origin, aims/purpose, participant number, study type, type of edema, diagnostic tools, major findings, and limitations of the study. The two investigators clarified and discussed any ambiguities or issues they encountered during the process of data extraction.

Synthesis of the Results

The studies were grouped by the type of clinical measurement tool used to assess all three types of edemas. These methods include US, LSG, CT, BIS, TDC, and MRI. We summarized these tools as they apply to each type of edema we were analyzing, i.e., lower extremity edema, lymphedema, or lipedema, considering data relevant to each measurement tool’s degree of complexity, cost, duration, and reliability.

Results

The following are the main results.

Lymphoscintigraphy

LSG is a minimally invasive imaging method used to map the lymphatic system and is effective at identifying lymphedema [21]. LSG provides functional information on the alterations of lymphatic flow, including but not limited to dermal backflow, flow obstruction, and decreased lymph node count. The method is reported to be safe, reproducible, well-tolerated, and easily applied [27-29].

Method: LSG provides images of the lymphatic vessels by injecting the radiotracer 99mTc albumin nanocolloid intradermally between the toes of both feet, which serves as a contrast agent for visualization [21]. The tracer is transported through the lymphatic drainage system into regional lymph nodes [30]. The resulting images show the dye moving through the lymphatic system, highlighting any obstructions present that might have caused the swelling [21]. Furthermore, LSG provides insight into both superficial and deep lymphatic systems [27]. Clinically, this method assists in lymphedema diagnosis in unclear cases, risk assessment, outcome prediction, and treatment evaluation [28].

Applications and outcomes: LSG has various methods of interpretation in the clinical field both quantitatively and qualitatively. Qualitative methods to identify lymph circulation abnormalities include visually identifying the presence of lymph, the presence of dermal backflow, or disruption of vasculature present in the resulting image [21]. Using qualitative methods, LSG is able to diagnose a dysfunction of the lymphatic system with a high specificity reaching 97-100% [20]. However, the sensitivity varies greatly due to significant variability in the lymph circulation (61-97%) [21]. On the other hand, quantitative methods measure the time the tracer travels through the lymphatic system or assess the regional uptake of the tracer in nearby lymph nodes [21].

In an effort to improve the sensitivity of LSG in diagnosing lymphedema of the lower extremities, several studies have reported the importance of combining both quantitative and qualitative methods of LSG evaluation [29]. While LSG is mainly used to diagnose lymphedema, it is unable to distinguish between lymphedema and lipedema. Lymph alterations were seen in lipedema as well, preventing the exclusion of lipedema as a diagnosis. On the other hand, the absence of lymph alterations does not confirm a lipedema diagnosis [30].

Furthermore, LSG cannot evaluate the clinical stage and grade of lipedema, as the LSG findings were not statistically significant [30]. Another drawback of LSG is the need to inject the patient with a contrast agent for visualization. Only the lymphatic nodes and vessels that specifically uptake the contrast can be seen, resulting in information restricted to these lymphatic structures only [27]. LSG also has a limited range and quality of view, as the images are low resolution [27]. Other modalities provide a better anatomical view of the lymphatic system [28].

Magnetic Resonance Imaging Approaches

MRI is useful for diagnosing and differentiating between lymphedema and lipedema by providing detailed anatomical and functional information, including the detection of lymphatic abnormalities, assessment of derangements in extremities, quantification of changes in lymphedema, and aiding in early diagnosis and treatment planning. MRI has been deemed by all studies as safe and reproducible, with only a few contraindications [17,27].

Method: MRI is utilized to characterize tissue changes, such as fluid accumulation and swelling, within affected anatomical regions. Several MRI techniques have been developed to better visualize the lymphatic system, including traditional two-dimensional (2D) MRI, 3D MRI, and magnetic resonance lymphography (MRL). Unlike 2D MRI, which produces a series of stacked images or slices, 3D MRI acquires data across multiple planes simultaneously, allowing for more comprehensive anatomic information about lymphatic malformation and dysfunction [27]. MRL is a specialized imaging technique that specifically focuses on visualizing the lymphatic system, providing detailed images of lymphatic vessels and flow patterns [17]. MRL can encompass both contrast-enhanced MRL and non-contrast MRL [17]. For contrast-enhanced MRL, gadolinium-based agents are injected intradermally, subcutaneously, or intranodally. These agents will then drain into the lymphatic system and lymphatic nodes, highlighting lymphatic vessels and nodes and allowing for imaging of lymphatic pathways altered by pathology [3]. Non-contrast magnetic resonance lymphography (NCMRL) does not rely on gadolinium usage; instead, it employs T2-weighted turbo-spin-echo sequences to emphasize static or slow-moving fluid-filled anatomical structures while suppressing background tissues [17].

Applications and outcomes: One study found that non-contrast 3D MRI plays a significant role in detecting both superficial and deep lymphatic abnormalities, including impaired and/or stagnant lymph flow and dilated lymphatic channels [27]. MRI not only displayed considerable anatomical findings in patients with lymphatic impairments but also provided significant insights into the functional defects of the lymphatics on local structures and tissue composition in one examination [27,31]. This dual functionality of MRI can be contrasted with LSG, which only provides functional lymphatic information, failing to provide detailed anatomy.

Clinically, MRL is considered a valuable tool in distinguishing between lipedema and lipolymphedema [17,31]. Contrast-enhanced MRL is reported to be exceptionally useful in situations where the degree of lymphatic involvement remains uncertain during the initial clinical assessment, or when optimal treatment requires a precise definition of the lymphatic system [31]. Non-contrast MRL can also be used to identify increased subcutaneous fat tissue in lipedema, in addition to revealing epifascial (superficial to the fascial layers but deep to the dermis) fluid collections prominent in lipolymphedema [17,32].

MRI can quantify the changes of lymphedema with accuracy after it was found that the MRI-measured water area of subcutaneous tissues of calves was able to display 100% sensitivity to lymphedema [32]. The accuracy of interstitial fluid measurements is considered highly important when assessing the disease's severity and establishing a treatment plan for patients [17,32].

Ultrasound

Ultrasound is useful for diagnosing and differentiating between lymphedema and lipedema by detecting variations in subcutaneous tissue thickness and echogenicity, providing a noninvasive and cost-effective method for clinical evaluation and diagnosis. This method is noninvasive and does not use ionizing radiation [18,19].

Method: To obtain an ultrasound image, a probe is placed on the skin that emits high-frequency sound waves and receives their echoes reflected from tissues and organs to create real-time images consisting of an epidermal entrance echo layer, a dermal layer, and a non- or low-echogenic subcutaneous tissue layer [18].

Applications and outcomes: A 2013 study on ultrasound use in lymphedema reports increased subcutaneous tissue thickness and increased subcutaneous echogenicity, with the echogenicity finding being the most valuable to help with staging lymphedema according to the International Society of Lymphology (ISL) staging [18]. A 2019 study showed that lymphedema was associated with increased skin thickness and dermal hypoechogenicity in the lower extremities compared to lipedema or controls [19]. On the other hand, lipedema was associated with increased skin thickness and subcutaneous hypoechogenicity [19]. Thus, ultrasound measurements can be used to distinguish between lymphedema and lipedema in clinical practice [19]. It can also be used to objectively identify the stage of lymphedema, using subcutaneous echogenicity findings. In addition to staging, ultrasound provides an advantage over CT and MRI for patients who might otherwise be unable to undergo a CT or MRI due to obesity [19]. Ultrasound machines do not have aperture or weight limitations as CT and MRI scanners. This makes ultrasound more accessible and comfortable for obese patients, as there are no constraints related to the size or weight of the imaging equipment [19]. A 2021 study used these dermal and subcutaneous tissue thickness findings to propose a quantification method to clinically diagnose lipedema [33]. That study reported optimal cutoff values of dermal thickness to diagnose lipedema with ultrasound as the following: 8.4 mm for the lateral leg region, 11.7 mm for the pre-tibial region, and 17.9 mm for the thigh region [33]. Furthermore, duplex ultrasound, a specialized ultrasound designed for vascular structures, serves as the initial approach to identify the cause of lower extremity edema when it has no clear etiology based on the patient’s history and physical examination [6]. Duplex ultrasound has a sensitivity and specificity rate of over 90% for DVT and venous reflux, and can even identify other causes, including hematomas, effusions, and popliteal cysts [6].

Computed Tomography

Computed tomography is useful for diagnosing and evaluating lymphedema, lipedema, and lower extremity edema, demonstrating high specificity and sensitivity for lymphedema and providing valuable information on structural changes and volume measurements in post-treatment settings for gynecologic cancer patients [22]. Additionally, computed tomography venography (CTV) is particularly beneficial for diagnosing DVT as a cause of lower extremity edema [34].

Method: Computed tomography creates high-resolution, cross-sectional image slices, varying from different size intervals, focusing on one area of the body, without the injection of contrast [35]. On the other hand, CTV requires a catheter to be placed in the antecubital vein to inject contrast to visualize the veins. Scans are taken after a certain amount of time for the contrast to travel [34].

Applications and outcomes: A 2002 study reported that CT scans were highly specific and sensitive for lymphedema (93% and 100%, respectively) and lipedema (95% and 100%, respectively) [35]. A common factor looked at among three of the identified studies was honeycombing, defined as a subcutaneous CT finding of thickened interstitial tissue that overlaps and is polygonal-shaped [34]. Honeycombing on CT has a 100% specificity for lymphedema, and more precisely, chronic lymphedema, as seen in a later study [22,35] when staging lymphedema. It is noteworthy that honeycombing was not found in lipedema [35]. A subsequent study found that honeycombing was not a specific finding to lymphedema and was also seen in generalized lower extremity edema and cellulitis [34].

CTV is particularly useful in the differential diagnosis of lower extremity swelling in post-treatment gynecologic cancer patients, particularly with DVT, lymphoceles, and cancer recurrence [22]. It measures the volume of subcutaneous tissues in this unique population after procedures such as pelvic lymph node dissection and radiotherapy or during regular follow-ups assessing cancer management [22]. Another study of gynecologic cancer patients reported that CTV of the lower extremity is more useful than ultrasound in diagnosing DVT [23].

Tissue Dielectric Constant

**Figure 2:**
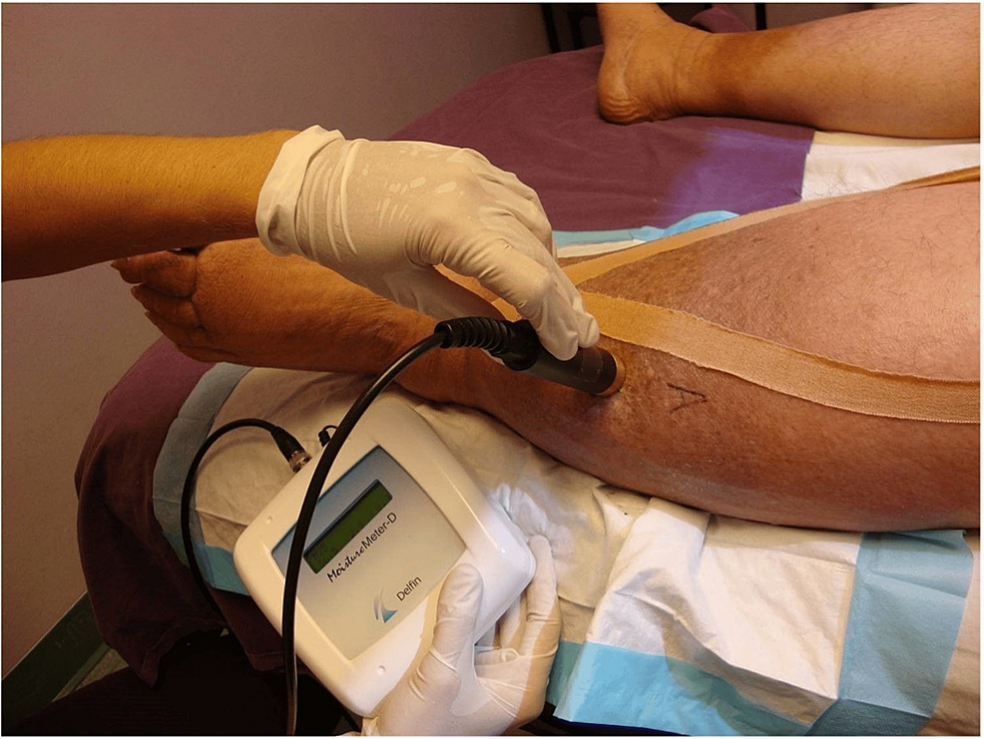
Tissue dielectric constant: lower extremity measurement illustration A combination of probe and control box is shown in use. A compact version that has the control elements incorporated into a single handheld device is also in use. The figure is courtesy of Dr. HN Mayrovitz.

TDC measurements are useful for diagnosing and evaluating lymphedema and edema in the lower extremities, providing a practical, cost-effective, and reliable method for assessing treatment efficacy, differentiating between various pathologies, and evaluating skin properties in patients with diabetes mellitus. Figure 2 illustrates its use in a patient.

**Figure 3:**
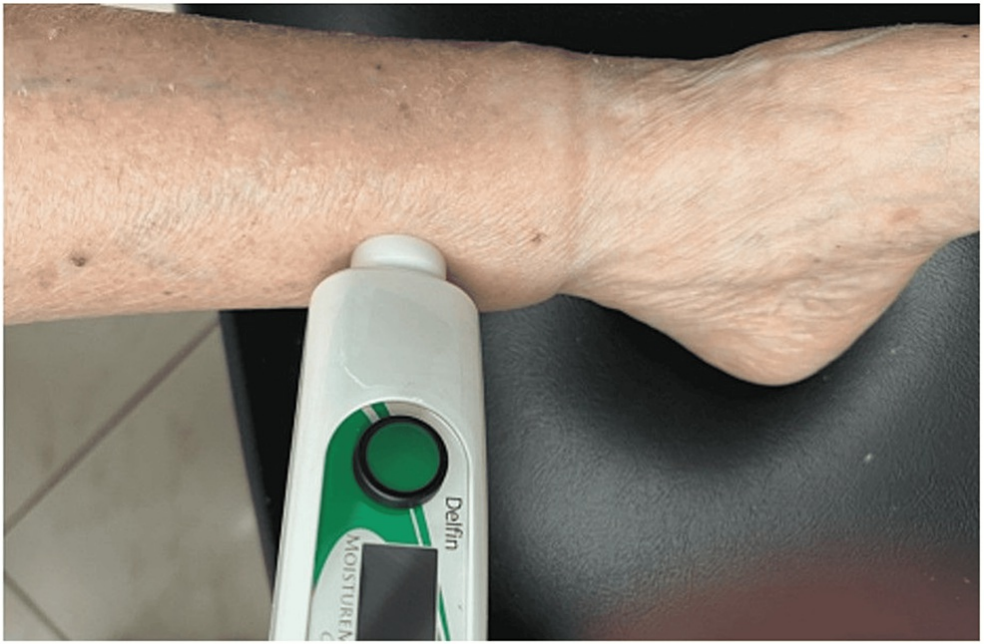
Application of compact device in lower extremity lymphedema The figure is courtesy of Dr. HN Mayrovitz.

Method: TDC measurements are a reliable and accurate way to measure localized skin tissue water content, both free and bound [36-38]. Pioneering research facilitated the creation of techniques for measuring the dielectric constants of tissues in vitro across different organs [39]. Currently, TDC can be measured via a compact, handheld, portable device and does not require users to be trained beforehand [25,36-38]. The probe of this device contacts the skin and takes four to five seconds to measure via a low-power electromagnetic signal transmitted into the skin [36-38]. In one version (as shown in Figure 2), the probe is connected to a control box that generates a 300 MHz signal, which it then transmits through the skin via an open-ended coaxial transmission line [36]. A segment of the signal partly reflects back, enabling the calculation of the complex reflection coefficient, which is used to determine the dielectric constant, defined as the ratio of tissue permittivity to vacuum permittivity [37,40]. The measurement at the desired skin location tends to be done in triplicate, with the average value used. Known TDC values from ethanol-water mixtures are typically used for calibrating the probes [40]. An example of a measurement with the handheld compact device is shown in Figure 3.

Applications and outcomes: The utility of TDC measurement was first demonstrated in the upper extremities. In association with lymphedema due to breast cancer treatment, TDC was shown to be the more valid method in diagnosing arm lymphedema, and total body fat percentage and body mass index (BMI) were unlikely to influence forearm TDC values [41,42]. Various studies have since applied this method to lower extremities using ratios of TDC measurements of lower extremity to upper extremity. One study used TDC ratios of foot/forearm and leg/forearm to develop values that can be used as thresholds for TDC measurements in the lower extremity. When applied in the clinical setting, values exceeding these thresholds would likely indicate the presence of edema and serve as an initial quantitative value to assess how edema presents in various pathologies [43]. Another study also developed a set of reference TDC values by utilizing lower-to-upper extremity ratio measurements, with the goal of decreasing TDC value variation among individuals using a practical and quick method. These ratios could be used when determining the presence of lymphedema and changes with treatment [2].

TDC measurements have also been reported as useful to assess lower extremity edema. When using TDC to differentiate between lymphedema and lipedema in women with chronic swelling of their legs, it was reported that mean TDC values of the ankle, lower leg, and foot for untreated lymphedema were higher and statistically significant compared to healthy controls, participants with lymphedema treated with compression bandaging, and participants with lipedema [25]. One study that looked at the therapeutic effect of a specific treatment for lymphedema, called complex decongestive physiotherapy (CDP), used TDC and circumference measurements as two metrics for evaluating CDP’s efficacy. It was concluded that TDC was the easier, quicker, and more practical measurement technique [44]. Another study evaluated lower leg localized skin water in subjects with a wide range of body mass index (BMI) and reported that no significant relationship was found between TDC and BMI [45].

TDC can be used as a reliable method to detect changes in lower limb lymphedema measurements following treatment with high test-retest reliability and acceptable measurement errors [46,47]. However, high inter-observer agreement was seen when performing TDC in ankle and lower leg measurements, but not in measuring foot values [25]. Additionally, measuring localized tissue water changes with TDC compared to girth measurements showed that TDC could be more sensitive to localized lymphedema changes and therefore is a useful complementary method and also an independent method of assessing lymphedema and treatment efficacy [48]. Furthermore, TDC can be used as a modality to evaluate the skin properties of patients with diabetes mellitus as TDC has been shown to have no relationship with glycosylated hemoglobin (HbA1c) or fasting glucose [49]. Additionally, TDC was able to detect increased, previously unrecognized fluid content in the foot dorsum. TDC values were significantly greater in diabetic patients compared to non-diabetic patients (P < 0.05) [50]. Overall, TDC can be used as a cost-effective diagnostic tool to aid in the initial evaluation of swollen legs in the clinical setting. Using TDC requires no expert training and reduces the need to use other expensive assessment methods such as CT or MRI [25]. Because preclinical lower extremity edema is often seen as a precursor to diabetic foot complications, using TDC can help prevent the progression of this illness to foot ulcers and neuropathy [50]. Also, TDC can be used in highly obese patients, which is a common problem in patients presenting with leg swelling [25].

Bioelectrical Impedance Spectroscopy

Bioelectrical impedance spectroscopy is a useful, noninvasive method to assess lower extremity lymphedema. When integrated with other diagnostic approaches, BIS enhances diagnostic accuracy by distinguishing lymphedema from other forms of swelling, aiding in early-stage detection and monitoring mild cases.

Method: This is a non-invasive technique that measures the electrical impedance of a limb at multiple frequencies under the assumption that reduced impedance indicates more fluid in the limb [51,52]. It is a widely used method in some clinics and extensive measurements have helped develop reference values.

Applications and outcomes: While a diagnosis of lymphedema is commonly made based on a patient's medical history and physical examination, incorporating BIS measurements into the initial evaluation may improve diagnosis and subsequent follow-up measurements may aid in tracking treatment progress [51,52]. It has been reported that these measurements have the potential to identify lymphedema in its early stages with high sensitivity and can therefore be a valuable tool for diagnosing patients with mild cases of the condition [51]. Based on this, this feature could be useful to detect small differences in leg volume that are sometimes challenging if traditional diagnostic methods are used alone [51]. A later study, however, reported that although BIS had a 100% specificity, its sensitivity was only 64%, and therefore, was not sensitive enough to rule out lymphedema confidently if there was a negative result, especially in early-stage disease [52]. In a different study, BIS was reported to have excellent reliability for use in all patients with unilateral and bilateral lower extremity lymphedema and be used to assess treatment impacts in individuals with mild to moderate lymphedema [46]. In this study, it was emphasized that this method’s reliability in more severe cases of lymphedema is in question because of the presence of skin changes and fibrosis.

BIS has limitations that can make diagnosis difficult. One study identified that BIS cannot quantify other tissue elements that increase lymphedema, such as fibrous and adipose tissue deposition [51]. Additionally, localized changes may be challenging to detect as BIS compares extracellular fluid (ECF) in one entire limb to the entire contralateral limb [2]. This also renders BIS unable to consider bilateral disease [52]. Lastly, extracellular fluid can be easily manipulated by temperature, compression, and daily activities, which may alter BIS results. As lymphedema progresses further, the limb can become more fibrotic leading to a reduction in tissue compliance. This may result in less fluid fluctuation, making BIS evaluation difficult [52].

Discussion

The purpose of this review was to describe the various clinical methods used to measure lower extremity edema, lymphedema, and lipedema, to explain their main application targets, and to provide some guidance on the advantages and disadvantages of each method.

Lower Extremity Edema

Duplex ultrasound is frequently used as the initial imaging modality for individuals experiencing swollen lower limbs without evident cause as identified through history, physical examination, or laboratory tests [6]. Based on a 2020 article providing a systematic approach to evaluating patients with leg swelling, the first and often only test utilized in the clinical setting for lower extremity edema with unknown etiology tends to be duplex ultrasound [6]. For further evaluation of edema, CTV was reported valuable in the gynecologic cancer patient population, specifically in diagnosing or ruling out DVT. It was preferred over ultrasound [22,23]. Five studies identified in this review utilized TDC in diagnosing lower extremity edema and have provided TDC value thresholds as a guide for diagnosis [40,43,45,49,50]. TDC measurement devices are readily available in the clinical setting, are less expensive than other imaging systems, and require little training to be performed successfully [25]. The literature reported TDC as useful in diabetic and obese patients, specifically where lower extremity swelling is more challenging to identify and diagnose [49,50]. This association between skin properties and diabetes using TDC, combined with the aforementioned characteristics, may make it the modality of choice for these conditions.

Lower Extremity Lymphedema

There are more studies dealing with the clinical assessment of lower extremity lymphedema than for edema or lipedema. Methods described include LSG, MRI, CT, US, TDC, and BIS. The present review indicates that LSG is the overall imaging modality of choice as it is the most thoroughly researched diagnostic method for lymphedema. Since its initial introduction, LSG has been considered the gold standard for diagnosing lymphedema and assessing the results of treatment. This consensus continues to be supported by recent literature [21,28,29].

LSG has been reported as easily applied, minimally invasive, safe, reproducible, and well-tolerated [27-29]. When opting to use LSG for lymphedema diagnosis, quantification, and qualification methods have been established in the literature to guide the interpretation of the findings [21,29]. Additionally, LSG has a reported sensitivity between 66% and 100% and a specificity between 83.5% and 99% [28]. In mild cases of lymphedema, LSG has been suggested as the modality of choice [28].

Some disadvantages uncovered by this review include LSG’s inability to distinguish between lymphedema and lipedema, its limitation to lymph structure imaging only, its low resolution, and its need for radiotracer injection [27,28]. Therefore, if greater anatomical details are desired for clinical use, cross-sectional imaging techniques such as CT and MRI should be used in conjunction with LSG [28]. MRI has excellent sensitivity, provides both anatomical and functional lymph information, and can differentiate between lymphedema and lipedema [27,31,32]. MRI is also safe, reproducible, and noninvasive [17,27,31,32]. While MRI could be more beneficial than LSG in diagnosing lymphedema, it should be noted that there is currently limited research on this modality, especially as the literature did not highlight the cost analysis of MRI. CT was also found to be sensitive and specific for lymphedema and can distinguish between lymphedema and other lower leg edema pathologies [34,35].

Although fewer studies in this review used US as the diagnostic modality for lymphedema, US is more cost-effective compared to LSG, MRI, and CT [18,19]. This makes it a practical choice for follow-up examinations in the management of lymphedema as it is readily available in many clinics.

Other noninvasive and cost-effective modalities are TDC and BIS. While the literature supports TDC as a reliable measurement tool in the early detection of lymphedema, it is particularly useful in assessing treatment efficacy and practical in monitoring edema changes over time [2,45-48]. One study demonstrated the potential usefulness of TDC in differentiating between lymphedema and lipedema in women presenting with swollen legs; however, further research on a larger population is necessary [25]. While TDC measurements are generally reliable in healthy men and women, TDC points close to bone and tendons in men should be used with caution as they have shown higher measurement errors and lower reliability [47]. One study discovered that BIS could identify lymphedema in its early stages by detecting differences in extracellular fluid and it can be used to track clinical changes in lymphedema after treatment [24,51]. However, it has been reported to have a high rate of false negatives and a sensitivity of 64% [52]. Despite this potential limitation, BIS is still widely used alone and in conjunction with more sensitive diagnostic modalities [24,52].

Lower Extremity Lipedema

While there is not a single definitive diagnostic test for lipedema, several methods are commonly used to aid in the diagnosis, including US, MRL, TDC, and LSG. High-frequency US imaging can be used to visualize subcutaneous adipose tissue and assess the thickness and characteristics of fat layers. The literature highlights the use of simple and reproducible US cutoff values to be used in diagnosing lipedema in the lower limbs [33].

Like US, MRL can provide detailed imaging of soft tissues and is useful for assessing the distribution and characteristics of adipose tissue [27,31,32]. The use of contrast-enhanced MRL can be limited in patients with contraindications to contrast injections. In these cases, non-contrast MRL can be used as an alternative in case of gadolinium hypersensitivity, helping in the differential diagnosis of lipedema [17,27,31,32]. Thus, both MRL and US can help differentiate lipedema from other conditions and may be valuable in surgical planning. While not commonly used for routine diagnosis of lipedema, LSG may be employed in cases where there is suspicion of lymphatic involvement or to rule out other conditions [30]. It can also aid in distinguishing between lipedema and lipolymphedema [30]. While there is limited literature regarding the use of TDC specifically for diagnosing lipedema, it was reported to be a valuable tool for differentiating lipedema from lymphedema in women with chronic swelling of their lower extremities [25]. TDC also requires minimal training to perform and is inexpensive compared with other imaging modalities [25].

Study uniqueness

To our knowledge, this is the first scoping review focusing on comparing the advantages and disadvantages of using these six measuring modalities for these causes of lower extremity swelling (edema, lymphedema, lipedema) in the clinical setting. Until recently, previous research had mainly focused on describing each method separately or at the most comparing two modalities or two edema types. The present review highlights the differences in these diagnostic modalities, each with a varying degree of complexity, cost, duration, validity, and reliability. It is hoped that this information will help guide in the process of selecting the optimal diagnostic tool tailored to each patient individually and to each type of lower extremity edema specifically.

Study limitations

Strengths of this study include a thoughtfully crafted search strategy, a meticulously executed scoping protocol, and a concentration on an under-researched subject matter pertaining to diagnostic tools for lymphedema, lipedema, and lower limb edema. However, this study is not without its limitations. Many studies we analyzed reported a small sample size and a limited number of patients as a limitation [17,23,46,50]. A few studies also reported unintentional selection bias due to the method of sampling patients, so not all stages of edema were considered [22,23,52]. The patient population in one study was primarily composed of individuals who sought medical attention following surgical management of lymphedema, potentially introducing selection bias regarding patient demographics and the severity of their condition [52]. Another study suggested that its retrospective nature may have contributed to selection bias [23]. Other researchers reported challenges in the consistency of BIS measurements and an overlap in impedance ratios [3,52]. Furthermore, in some cases, certain diagnostic techniques have not been evaluated for their effectiveness in diagnosing different types of edemas, which has made comparisons and assessments challenging. This lack of research into certain diagnostic modalities has led to the absence of a definitive test to accurately diagnose edemas [17,30]. Although there are limitations in both our scoping review process and the existing literature, the information presented is still valuable given the under-researched nature of this field, and the need for new insights to improve patient care.

## Conclusions

This review aimed to compile relevant and available information about clinical assessment modalities for lower extremity edema, lymphedema, and lipedema, and report their benefits and drawbacks. Such information housed in a single widely available document was thought to be potentially useful as an information source for those involved in the diagnosis or treatment of these conditions.

Findings with respect to lower extremity edema with unknown etiology indicated that the most frequently used initial modality is duplex ultrasound based on its high sensitivity and specificity for DVTs and venous reflux. Findings with respect to the effective diagnosis of lower extremity lymphedema indicated the efficacy of lymphoscintigraphy despite it being minimally invasive. Findings with respect to routine noninvasive detection and tracking of lower extremity lymphedema indicate the use of TDC in part due to its localized assessment capabilities and ease of use and BIS in part due to its wide use despite its reported low-to-moderate sensitivity. Findings with respect to diagnosis and tracking of lower extremity lipedema indicated that the most frequently used modalities are ultrasound and magnetic resonance lymphoscintigraphy with the former being more cost-effective and sometimes more accessible than the latter. Findings with respect to the differential diagnosis between lymphedema and lipedema indicated that MRI is the most frequently used modality as it provides comprehensive anatomical and functional information, including imaging of lymphatic irregularities and tissue changes, and can aid in early diagnosis. However, ultrasound is one pragmatic alternative for use with obese patients or when the availability or use of MRI is not an option.
